# Altered peptide ligands inhibit arthritis induced by glucose-6-phosphate isomerase peptide

**DOI:** 10.1186/ar2854

**Published:** 2009-11-09

**Authors:** Keiichi Iwanami, Isao Matsumoto, Yohei Yoshiga, Asuka Inoue, Yuya Kondo, Kayo Yamamoto, Yoko Tanaka, Reiko Minami, Taichi Hayashi, Daisuke Goto, Satoshi Ito, Yasuharu Nishimura, Takayuki Sumida

**Affiliations:** 1 Department of Clinical Immunology, Doctoral Program in Clinical Science, Graduate School of Comprehensive Human Science, University of Tsukuba, 1-1-1 Tennoudai, Tsukuba 305-8575, Japan; 2 PRESTO, Japan Science and Technology Agency, 4-1-8 Honcho Kawaguchi, Saitama 332-0012, Japan; 3 Department of Immunogenetics, Graduate School of Medical Sciences, Kumamoto University, 2-39-1 Kurokami, Kumamoto 860-8556, Japan

## Abstract

**Introduction:**

Immunosuppressants, including anti-TNFα antibodies, have remarkable effects in rheumatoid arthritis; however, they increase infectious events. The present study was designed to examine the effects and immunological change of action of altered peptide ligands (APLs) on glucose-6-phosphate isomerase (GPI) peptide-induced arthritis.

**Methods:**

DBA/1 mice were immunized with hGPI_325-339_, and cells of draining lymph node (DLN) were stimulated with hGPI_325-339 _to investigate the T-cell receptor (TCR) repertoire of antigen-specific CD4^+ ^T cells by flow cytometry. Twenty types of APLs with one amino acid substitution at a TCR contact site of hGPI_325-339 _were synthesized. CD4^+ ^T cells primed with human GPI and antigen-presenting cells were co-cultured with each APL and cytokine production was measured by ELISA to identify antagonistic APLs. Antagonistic APLs were co-immunized with hGPI_325-339 _to investigate whether arthritis could be antigen-specifically inhibited by APL. After co-immunization, DLN cells were stimulated with hGPI_325-339 _or APL to investigate Th17 and regulatory T-cell population by flow cytometry, and anti-mouse GPI antibodies were measured by ELISA.

**Results:**

Human GPI_325-339_-specific Th17 cells showed predominant usage of TCRVβ8.1 8.2. Among the 20 synthesized APLs, four (APL 6; N329S, APL 7; N329T, APL 12; G332A, APL 13; G332V) significantly reduced IL-17 production by CD4^+ ^T cells in the presence of hGPI_325-339_. Co-immunization with each antagonistic APL markedly prevented the development of arthritis, especially APL 13 (G332V). Although co-immunization with APL did not affect the population of Th17 and regulatory T cells, the titers of anti-mouse GPI antibodies in mice co-immunized with APL were significantly lower than in those without APL.

**Conclusions:**

We prepared antagonistic APLs that antigen-specifically inhibited the development of experimental arthritis. Understanding the inhibitory mechanisms of APLs may pave the way for the development of novel therapies for arthritis induced by autoimmune responses to ubiquitous antigens.

## Introduction

Rheumatoid arthritis (RA) is characterized by symmetrical polyarthritis and joint destruction. Although the etiology is considered autoimmune reactivity to some antigens, the exact mechanisms are not fully understood. Pathological examinations show that most of the lymphocytes infiltrating the synovium in RA are CD4^+ ^T cells, which can recognize some antigens and expand oligoclonally intraarticularly [1]. These findings imply the possible role of CD4^+ ^T cells in the pathogenesis of RA. Previous studies showed that cytotoxic T-lymphocyte antigen-4 immunoglobulin and tacrolimus have remarkable effects on RA, and stressed the importance of CD4^+ ^T cells in the pathogenesis of RA [2-4].

Although the exact helper T-cell lineage critical in RA remains elusive, previous animal studies reported that Th17 cells play a crucial role and that Th1 cells may have a protective role against the progress of arthritis in most mouse models with the exception of proteoglycan-induced arthritis in Balb/c mice [5]. Collagen-induced arthritis in the C57BL/6 background is markedly suppressed in IL-17-deficient mice [6], and glucose-6-phosphate isomerase (GPI)-induced arthritis in the DBA/1 background and antigen-induced arthritis in the C57BL/6 background are also suppressed by the administration of anti-IL-17 antibodies (Abs) [7,8]. In these models, complete Freund's adjuvant is used for the induction of arthritis; therefore it is possible that the components of *Mycobacterium tuberculosis *affect the cytokine dependency. The arthritis seen in IL-1 receptor antagonist-deficient mice in the Balb/c background and SKG mice in the Balb/c background, however, is completely suppressed in IL-17-deficient mice [9,10]. These findings indicate that Th17 cells play a central role in murine models independent of mouse strains and target antigens.

IL-17 is also considered to play a crucial role in host defense. IL-17 signaling seems essential for the recruitment of neutrophils to the alveolar space in pneumonia caused by *Klebsiella pneumoniae*, *Mycoplasma pneumoniae *and *Pneumocystis jiroveci *[11-13]. IL-17 is also involved in mucosal host defense against oropharyngeal candidasis via salivary antimicrobial factors, in addition to neutrophil recruitment [14]. Furthermore, IL-17 production by γδ T cells is essential against peritonitis caused by *Escherichia coli *[15]. In this regard, anti-cytokine therapies such as infliximab and tocilizumab have been applied to clinical treatment and have shown striking effects on RA [16-19]; anti-IL-17 therapy could therefore be useful in the treatment of RA. Blockade of IL-17 could increase the likelihood of infections, however, and the use of such a strategy would be limited just like the case of infliximab and tocilizumab.

Altered peptide ligands (APLs) are peptides with substitutions in amino acid residues at T-cell receptor (TCR) contact sites, and can be either agonistic, antagonistic with partial activation or antagonistic [20]. These three different actions seem to depend on the site and residue of the peptide substitution [21]. The antagonistic APLs can inhibit the function of limited T-cell populations, and thus they could be potentially useful as antigen-specific therapy for autoimmune diseases in which T cells play a pathogenic role. Indeed, APLs have been proven effective in the suppression of several autoimmune models. In an arthritis model, previous studies identified type II collagen CII_245-270 _as a prominent T-cell epitope in collagen-induced arthritis in DBA/1 mice, and found that co-immunization with the analog peptide (I260A, A261B(hydroxyproline), F263N), also known as A9, significantly suppressed the disease [22,23]. As reported previously, however, the type II collagen residues CII 260 (I) and CII 263 (F) are I-Aq (MHC class II of DBA/1 mice) binding sites, and A9 was confirmed not to bind I-Aq molecules [24,25]. The analog peptide A9 therefore seems to differ from conventional APLs, and the inhibitory effect and the mechanisms of conventional APLs on arthritis remain to be defined.

Several models of arthritis have so far been described and analyzed to understand the etiological mechanisms of RA. GPI-induced arthritis, a murine model of RA, is induced by immunization of DBA/1 mice with recombinant human GPI (rhGPI) [26]. We demonstrated previously that the Th17 subset of CD4^+ ^T cells played a central role in the pathogenesis of GPI-induced arthritis; GPI-specific CD4^+ ^T cells were skewed to T_H_-17 at the time of onset, and blockade of IL-17 resulted in a significant amelioration of arthritis [7]. We have also demonstrated that the major epitope of CD4^+ ^T cells in GPI-induced arthritis was hGPI_325-339_, and immunization with the peptide induced severe polyarthritis (GPI peptide-induced arthritis) [27].

The present work is an extension to the above studies. Specifically, we explored the antigen-specific Th17 inhibition, explored the effects of APLs on arthritis, and investigated the inhibitory mechanisms of APLs, using the T-cell-dependent model of GPI peptide-induced arthritis. The results showed that many hGPI_325-339_-specific CD4^+ ^T cells employed Vβ8.1 8.2 as the TCR repertoire, and co-immunization with APL (N329S, N329T, G332A, or G332V) significantly inhibited the development of arthritis. Our analysis of the inhibitory mechanisms of APLs indicates that our APLs can function as TCR antagonists; however, they can differentiate naïve CD4^+ ^T cells to Th17 cells, but not Th2 cells or regulatory T cells. Based on these findings, we define a new aspect for APLs, and propose that they may provide the basis for the invention of new antigen-specific therapy.

## Materials and methods

### Mice

DBA/1 mice were purchased from Charles River Laboratories, Yokohama, Japan. All mice were kept under specific pathogen-free conditions, and all experiments were conducted in accordance with the institutional ethical guidelines.

### Glucose-6-phosphate isomerase and synthetic peptides

The rhGPI and recombinant mouse GPI were prepared as described previously [28,29]. Briefly, human or mouse GPI cDNA was inserted into the plasmid pGEX-4T3 (Pharmacia, Uppsala, Sweden) for expression of glutathione-*S*-transferase-tagged proteins. *E. coli *harboring pGEX-hGPI plasmid was allowed to proliferate at 37°C, before the addition of 0.1 mM isopropyl-β-d-thiogalactopyranoside to the medium, followed by further culture overnight at 30°C. The bacteria were lysed with a sonicator and the supernatant was purified with a glutathione-sepharose column (Pharmacia). The purity was estimated by SDS-PAGE.

Peptides for screening were synthesized with 70% purity by Wako Pure Chemical Industries, Ltd (Osaka, Japan), and peptides of a major peptide and antagonistic altered peptide ligands were synthesized with 90% purity by Invitrogen (Carlsbad, CA, USA). OVA_323-339 _peptide was purchased from AnaSpec (San Jose, CA, USA).

### Induction of arthritis

DBA/1 mice were immunized with 10 μg synthetic peptides for GPI peptide-induced arthritis in complete Freund's adjuvant (Difco Laboratories, Detroit, MI, USA), and in the indicated experiments 50 μg altered peptide ligands were used with 10 μg GPI_325-339 _for co-immunization. The synthetic peptides were emulsified with complete Freund's adjuvant at a 1:1 ratio (v/v). For induction of arthritis, 150 μl emulsion was injected intradermally at the base of the tail, and 200 ng pertussis toxin was injected intraperitoneally on days 0 and 2 after immunization.

The arthritis score was evaluated visually using a score of 0 to 3 for each paw. A score of 0 represented no evidence of inflammation, 1 represented subtle inflammation or localized edema, 2 represented easily identified swelling that was localized to either the dorsal or ventral surface of the paws, and 3 represented swelling in all areas of the paws.

### Screening of antagonistic altered peptide ligands

Mice were sacrificed on the indicated day. Spleens and draining lymph nodes (DLNs) were harvested, and splenocytes were hemolyzed with a solution of 0.83% NH_4_Cl, 0.12% NaHCO_3 _and 0.004% EDTA_2_Na in PBS. Single-cell suspensions were prepared in RPMI 1640 medium (Sigma-Aldrich, St Louis, MO, USA) containing 10% fetal bovine serum, 100 U/ml penicillin, 100 μg/ml streptomycin and 50 μM 2-mercaptoethanol. CD4^+ ^T cells from DLNs and CD11c^+ ^dendritic cells from spleens were isolated by magenetic-activated cell sorting (Miltenyi Biotec, Bergisch Gladbach, Germany). The purity of the collected cells (>97%) was confirmed by flow cytometry. CD11c^+ ^dendritic cells treated with 50 μg/ml mitomycin C were used as antigen-presenting cells (APCs). The purified CD4^+ ^T cells and APCs were co-cultured with 10 μM synthetic peptide at a ratio of 1:3 in 96-well round-bottom plates (Nunc, Roskilde, Denmark) at 37°C under 5% carbon dioxide for 72 hours. The supernatants were assayed for IL-10 and IL-17 by the Quantikine ELISA kit (R&D Systems, Minneapolis, MN, USA).

### Pre-pulse assay

The pre-pulse assay was conducted as described previously [30]. Briefly, CD11c^+ ^APCs from spleens (4 × 10^4^/well) were cultured with a suboptimal concentration of GPI_325-339 _(3 μM) for 2 hours. In the meantime, native peptides were loaded onto APCs and presented on MHC. After 2 hours of incubation, APCs were washed twice to remove unbound peptides, and 30 μM each antagonistic APL was added. After 18 hours of culture, CD4^+ ^T cells (2 × 10^4^/well) from DLNs were added, and after an additional 72 hours of culture the supernatants were assayed for IL-17 and IL-10 by ELISA. The inhibition ratio was calculated as follows:

1 − (IL-17 concentration in the presence of native peptides and APLs / IL-17 concentration in the presence of native peptides only) x 100 (%)

### Flow cytometry

Mice were sacrificed on the indicated day. The popliteal lymph nodes were harvested and single-cell suspensions were prepared as described above. Cells (1 × 10^6^/ml) were stimulated with 100 μg/ml rhGPI in 96-well round-bottom plates (Nunc) for 24 hours and GoldiStop (BD PharMingen, San Diego, CA, USA) was added for the last 2 hours of each culture. Cells were first stained extracellularly, fixed and permeabilized with Cytofix/Cytoperm solution (BD PharMingen) and then stained intracellularly. Regulatory T cells were stained with the Mouse Regulatory T cell Staining kit (eBioscience, San Diego, CA, USA) according to the protocol supplied by the manufacturer. For TCR repertoire screening, the Mouse TCR Screening Panel (BD PharMingen) was used. Samples were acquired on FACSCalibur (BD PharMingen) and data were analyzed with FlowJo (Tree Star, Ashland, OR, USA).

### Analysis of anti-glucose-6-phosphate isomerase antibody

Sera were taken from immunized mice on day 28 and were diluted 1:500 (for IgG, IgG_2a_, IgG_2b _and IgG_3_) or 1:8,000 (for IgG_1_) in blocking solution (25% Block Ace (Dainippon Sumitomo Pharma, Osaka, Japan) in PBS) for antibody analysis. We also prepared 96-well plates (Sumitomo Bakelite, Tokyo, Japan) coated with 5 μg/ml recombinant mouse GPI for 12 hours at 4°C. After washing twice with a washing buffer (0.05% Tween20 in PBS), the blocking solution was used for blocking nonspecific binding for 2 hours at room temperature. After two washes, 150 μl diluted serum was added and incubated for 2 hours at room temperature. After three washes, alkaline phosphatase-conjugated anti-mouse IgG, horseradish peroxidase-conjugated anti-mouse IgG_1_, IgG_2a_, IgG_2b _(Zymed Laboratories, San Francisco, CA, USA) or IgG_3 _(Invitrogen) was added at a final dilution of 1:5,000 for 1 hour at room temperature. After three washes, color was developed with substrate solution (1 alkaline phosphatase tablet (Sigma-Aldrich) per 5 ml alkaline phosphatase reaction solution (containing 9.6% diethanolamine and 0.25 mM MgCl_2_, pH 9.8)) or tetramethylbenzidine (KPL, Gaithersburg, MD, USA). Plates were incubated for 20 to 60 minutes at room temperature and the optical density was measured by a microplate reader at 405 nm (for IgG) or at 450 nm (for IgG_1_, IgG_2a_, IgG_2b _and IgG_3_).

### Statistical analysis

All data are expressed as the mean ± standard error of the mean or standard deviation. Differences between groups were examined for statistical significance using the Mann-Whitney U test. Differences of arthritis incidence between groups were examined using Fisher's exact test. *P *< 0.05 denotes the presence of a statistically significant difference.

## Results

### Designing and screening antagonistic altered peptide ligands

We reported previously that the major T-cell epitope in GPI-induced arthritis is hGPI_325-339_, and immunization with the peptide provokes symmetrical polyarthritis (GPI peptide-induced arthritis) [28]. To investigate the effects of APLs in GPI peptide-induced arthritis, we first designed APLs of hGPI_325-339_. Since the MHC binding sites of hGPI_325-339 _exist at P1 (I328), P4 (F331), and P7 (E334) (IWYINCFGCETHAML) [25,28], the amino acid residues of the TCR contact sites at P0 (Y327), P2 (N329), P3 (C330), P5 (G332), P6 (C333), and P8 (T335) were substituted for another peptide to design 20 types of APLs (Table 1).

**Table 1:** hGPI_325--339_-derived altered peptide ligands used in the present study

325 to 339	I	W	Y	I	N	C	F	G	C	E	T	H	A	M	L
APL 1	--	--	N		--	--	--	--	--	--	--	--	--	--	--
APL 2	--	--	Q		--	--	--	--	--	--	--	--	--	--	--
APL 3	--	--	S		--	--	--	--	--	--	--	--	--	--	--
APL 4	--	--	T		--	--	--	--	--	--	--	--	--	--	--
APL 5	--	--	--		Q	--		--	--		--	--	--	--	--
APL 6	--	--	--		S	--		--	--		--	--	--	--	--
APL 7	--	--	--		T	--		--	--		--	--	--	--	--
APL 8	--	--	--		--	N		--	--		--	--	--	--	--
APL 9	--	--	--		--	Q		--	--		--	--	--	--	--
APL 10	--	--	--		--	S		--	--		--	--	--	--	--
APL 11	--	--	--		--	T		--	--		--	--	--	--	--
APL 12	--	--	--		--	--		A	--		--	--	--	--	--
APL 13	--	--	--		--	--		V	--		--	--	--	--	--
APL 14	--	--	--		--	--		--	N		--	--	--	--	--
APL 15	--	--	--		--	--		--	Q		--	--	--	--	--
APL 16	--	--	--		--	--		--	S		--	--	--	--	--
APL 17	--	--	--		--	--		--	T		--	--	--	--	--
APL 18	--	--	--		--	--		--	--		N	--	--	--	--
APL 19	--	--	--		--	--		--	--		Q	--	--	--	--
APL 20	--	--	--		--	--		--	--		S	--	--	--	--

The MHC binding sites exist at glucose-6-phosphate isomerase (GPI) 328 (I), GPI 331 (F) and GPI 334 (E) as indicated (underlined). The amino acid residues of the T-cell receptor contact sites at P0 (Y327), P2 (N329), P3 (C330), P5 (G332), P6 (C333), and P8 (T335) were substituted for the indicated peptides to design 20 types of altered peptide ligands (APLs).

**Figure 1:**
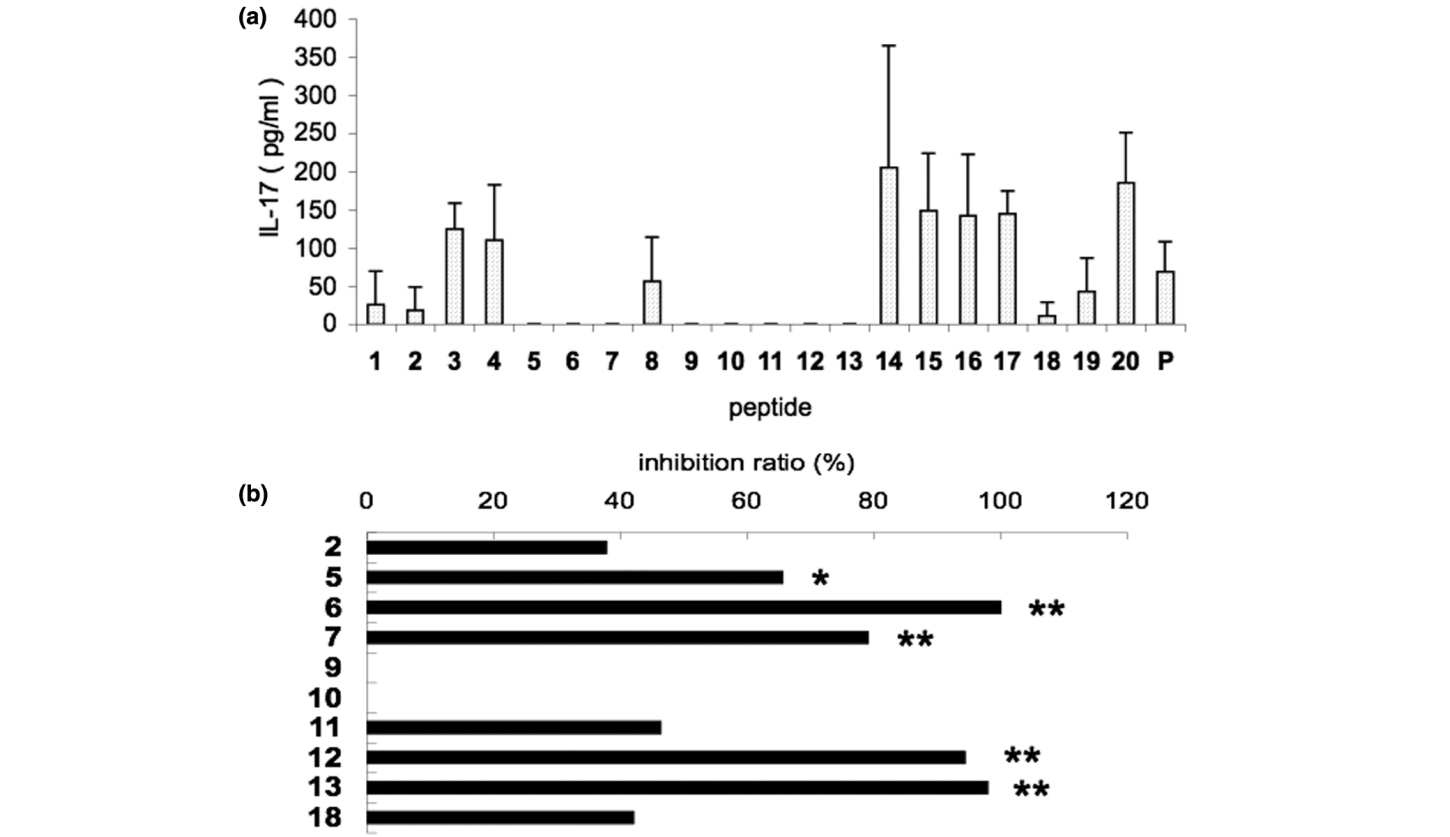
Altered peptide ligands markedly suppress IL-17 production by glucose-6-phosphate isomerase-primed CD4^+ ^T cells. Altered peptide ligand (APL) 6, APL 7, APL 9, APL 12 and APL 13 markedly suppress IL-17 production by glucose-6-phosphate isomerase (GPI)-primed CD4^+ ^T cells. Mice were sacrificed on day 8 after immunization. CD4^+ ^T cells were purified from draining lymph node cells of recombinant human GPI (hGPI)-immunized DBA/1 mice, and CD11c^+ ^antigen-presenting cells (APCs) were purified from spleen cells. **(a) **CD4^+ ^T cells primed with hGPI and APCs were co-cultured with 10 μM synthetic peptide for 72 hours. The supernatants were assayed for IL-17 by ELISA. P, positive control (hGPI_325--339_). **(b) **CD11c^+ ^APCs were cultured with a suboptimal concentration GPI_325--339_ (3 μM) for 2 hours, washed twice to remove unbound peptides, and 30 μM each antagonistic APL was added. After 18 hours of culture, CD4^+ ^T cells (2 × 10^4^/well) were added, and after an additional 72 hours of culture, the supernatants were assayed for IL-17 by ELISA. The inhibition ratio was calculated as stated in Pre-pulse assay. Data presented as average ± standard deviation of three culture wells. *P < 0.05, **P < 0.01 (Mann--Whitney U test). Representative data of two independent experiments.

To select antagonistic APLs, CD4^+ ^T cells primed with rhGPI and APCs were co-cultured with each APL. The IL-17 production was markedly lower with APL 2, APL 5, APL 6, APL 7, APL 9, APL 10, APL 11, APL 12, APL 13, and APL 18 than with hGPI_325-339_, and therefore these APLs were considered candidates of antagonistic APLs (Figure 1a). None of the APLs induced IL-4 and IL-10 production (data not shown). We next explored the potency of the APLs in inhibiting IL-17 production in the presence of hGPI_325-339_. In the pre-pulse assay, APL 6 (N329S), APL 7 (N329T), APL 12 (G332A), and APL 13 (G332V) significantly reduced IL-17 production by CD4^+ ^T cells primed with rhGPI in the presence of hGPI_325-339 _(*P *< 0.01) (Figure 1b). We therefore considered these four APLs as antagonistic APLs.

### Inhibition of arthritis by antagonistic altered peptideligands

**Figure 2:**
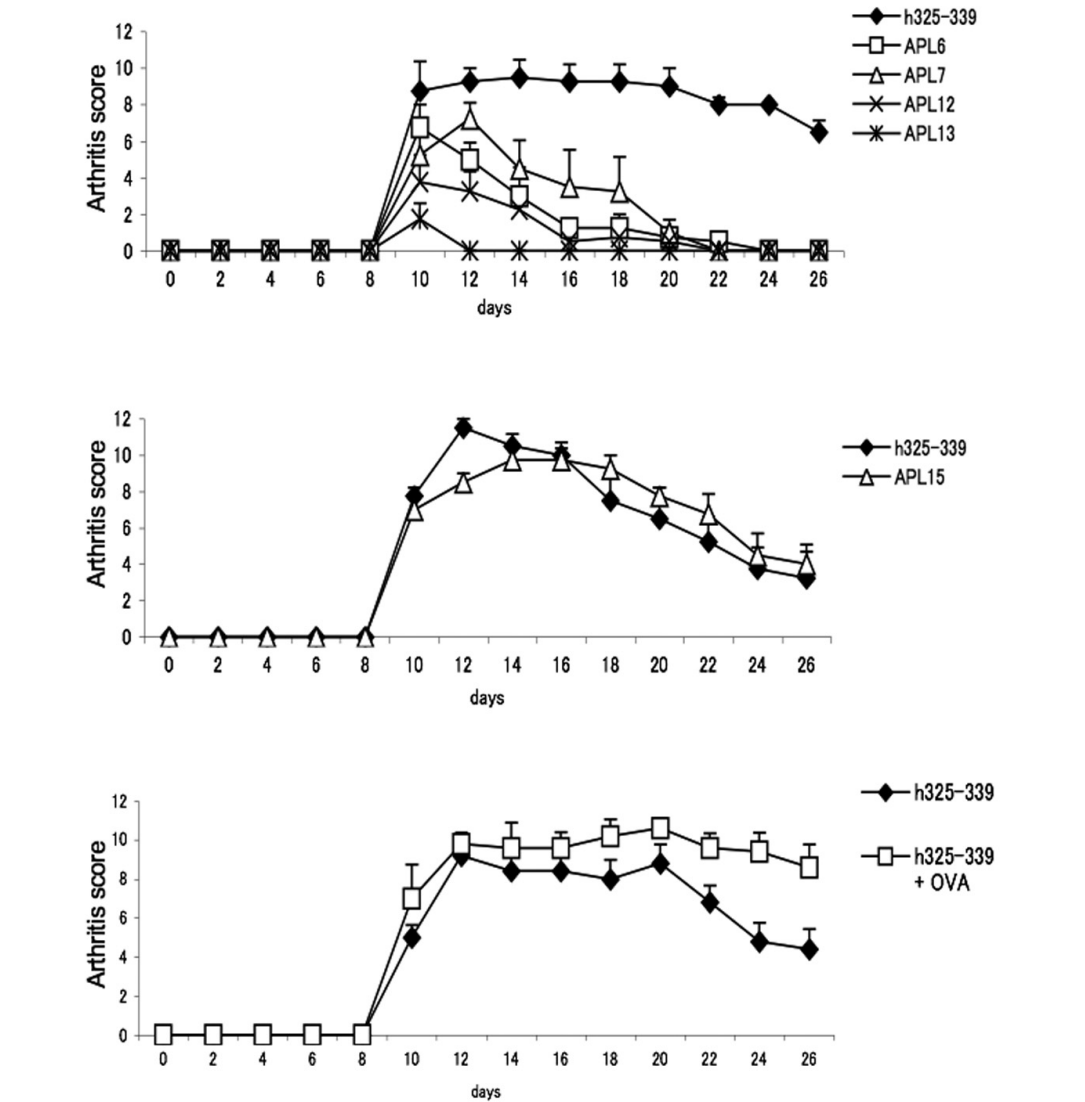
Co-immunization with antagonistic altered peptide ligands suppresses the development of arthritis. Mice were co-immunized with antagonistic altered peptide ligand (APL) 6, APL 7, APL 12, APL 13 (upper panel), the agonistic APL 15 (middle panel) or OVA peptide (lower panel). Progression of arthritis was significantly suppressed in mice co-immunized with APL 6, APL 12 and APL 13 after day 12, and in mice co-immunized with APL 7 after day 16 (*P *< 0.05, Mann--Whitney U test). Data presented as mean arthritis score (± standard error of the mean) of four mice in one representative experiment of at least two independent experiments.

Since GPI peptide-induced arthritis is mediated by Th17 and antagonistic APLs can suppress IL-17 production, we explored the efficacy of the prepared APLs on the inhibition of arthritis. First, we immunized DBA/1 mice with each APL alone, and confirmed that no overt arthritis developed (data not shown). DBA/1 mice were then co-immunized with hGPI_325-339 _and each APL to explore the development of arthritis. Mice co-immunized with APL 6, APL 12 and APL 13 developed significantly attenuated arthritis after day 12, and those co-immunized with APL 7 after day 16, compared with mice immunized with hGPI_325-339 _alone (*P *< 0.05) (Figure 2, upper panel). Co-immunization with APL 13 also significantly suppressed the incidence of arthritis (*P *< 0.05) (Table 2). Co-immunization with hGPI_325-339 _and APL 15, an agonistic APL, however, did not affect the severity or course of arthritis (Figure 2, middle panel). Moreover, mice co-immunized with hGPI_325-339 _and OVA_323-339 _also had a similar course of arthritis to hGPI_325-339 _alone (Figure 2, lower panel).

**Table 2:** Effects of co-immunization with altered peptide ligands on the development of arthritis

Co-immunization	Incidence	Day of onset	Maximum severity
None	8/8	10 ± 0.0	10.9 ± 1.4
APL 6	8/8	10 ± 0.0	7.5 ± 2.0*
APL 7	6/8	10 ± 0.0	7.8 ± 1.7*
APL 12	7/8	10.3 ± 0.8	5.0 ± 1.2**
APL 13	3/8†	10 ± 0.0	4.0 ± 0.0**

Mice were co-immunized with 10 μg glucose-6-phosphate isomerase hGPI_325--339_and 50 μg indicated antigen. Data presented as incidence or mean ± standard deviation. ^†^*P *< 0.05 (Fisher's exact test). *P < 0.005, **P < 0.001 (Mann--Whitney U test).

### Identification of TCRVβ usage of hGPI_325-339_-specificTh17 cells

**Figure 3:**
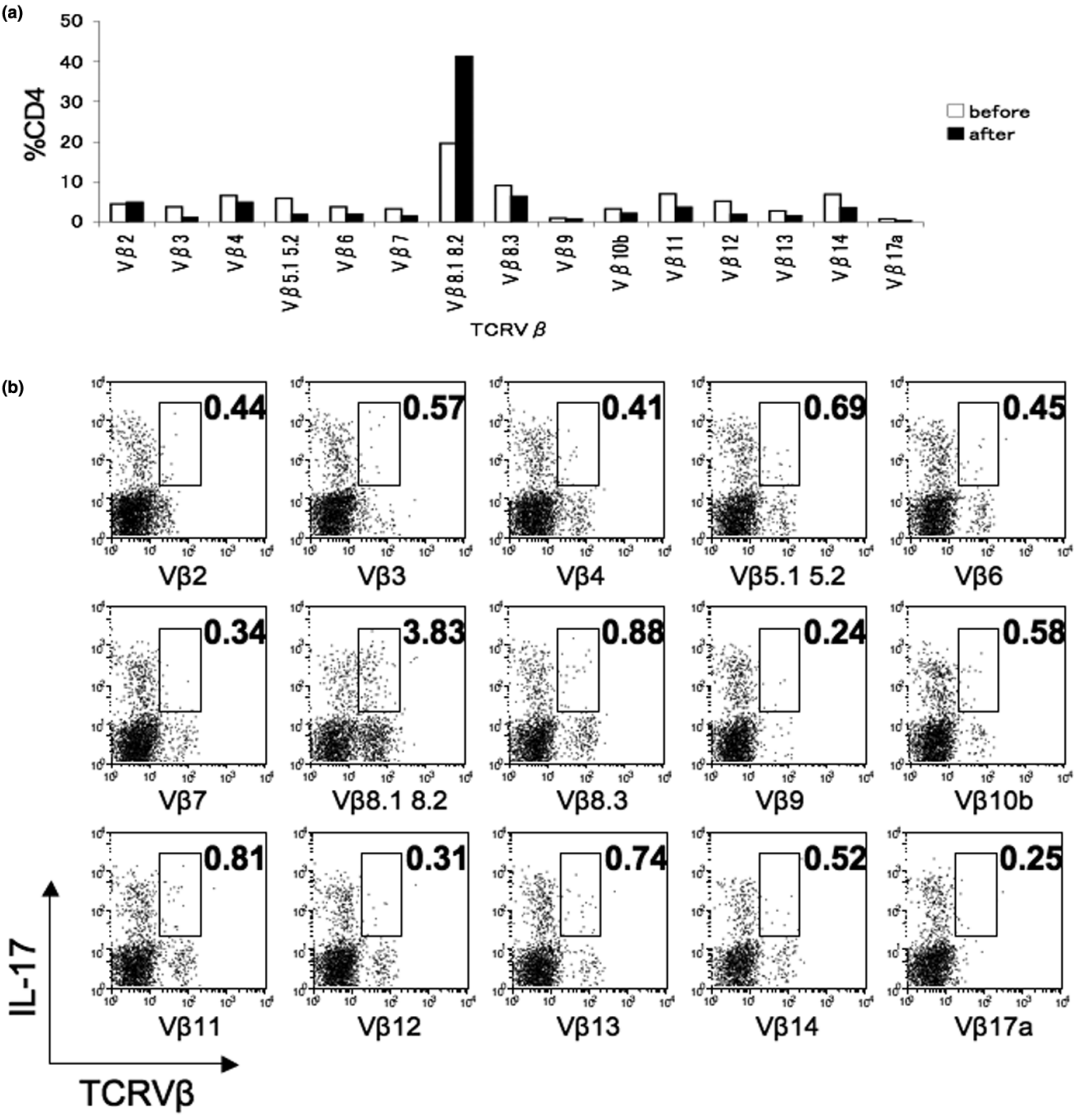
Human glucose-6-phosphate isomerase-specific Th17 cells use TCRVβ8.1 8.2. Many glucose-6-phosphate isomerase hGPI_325--339_-specific Th17 cells use TCRVβ8.1 8.2. Mice were immunized with 10 μg hGPI_325--339_, and draining lymph node cells on day 6 were stimulated with 20 μM hGPI_325--339 _*in vitro*. **(a) **Ratios of TCRVβ repertoire to CD4^+ ^T cells. The TCRVβ repertoire of CD4^+ ^T cells was analyzed by flow cytometry: before stimulation with hGPI_325--339 _*in vitro *for 72 hours, and after stimulation. **(b) **GoldiStop was added in the last 4 hours of the 24-hour culture. Flow cytometry analysis for IL-17 was gated in CD4^high ^cells. Representative data of two independent experiments.

To investigate the inhibitory mechanisms of the antagonistic APLs, we explored TCRVβ usage of hGPI_325-339_-specific CD4^+ ^T cells. The CD4^+ ^T cells primed with hGPI_325-339 _were stimulated with hGPI_325-339 _*in vitro *and the TCRVβ repertoire was analyzed by flow cytometry and compared with that before stimulation. Stimulation with hGPI_325-339 _expanded the population of CD4^+ ^T cells with TCRVβ8.1 8.2 (Figure 3a). We also found that much of IL-17 was produced by CD4^+ ^T cells with TCRVβ8.1 8.2 following stimulation with hGPI_325-339 _(Figure 3b). These data indicate that many hGPI_325-339_-specific Th17 cells use TCRVβ8.1 8.2.

### Effect of antagonistic altered peptide ligands on differentiation of Th17 and regulatory T cells

**Figure 4:**
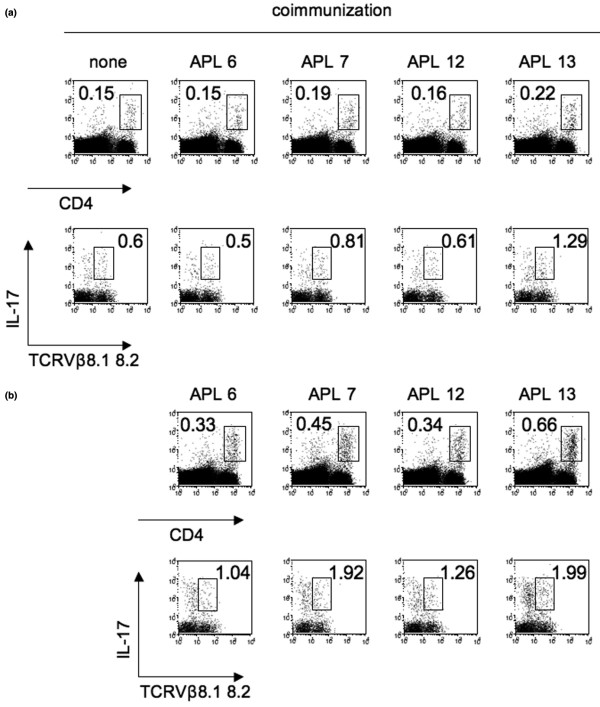
Co-immunization with altered peptide ligands does not affect IL-17 production. Mice were immunized with 10 μg glucose-6-phosphate isomerase hGPI_325--339_ and 50 μg each altered peptide ligand (APL). Draining lymph node cells on day 6 were stimulated for 24 hours *in vitro ***(a) **with 10 μM mouse GPI_325--339 _or **(b) **with 10 μM corresponding APL. GoldiStop was added in the last 4 hours of each culture. Flow cytometry analysis for IL-17 and TCRVβ repertoire was gated in CD4^high ^cells. None, immunization with no APL (hGPI_325--339_alone). Representative flow cytometry data of two independent experiments.

**Figure 5:**
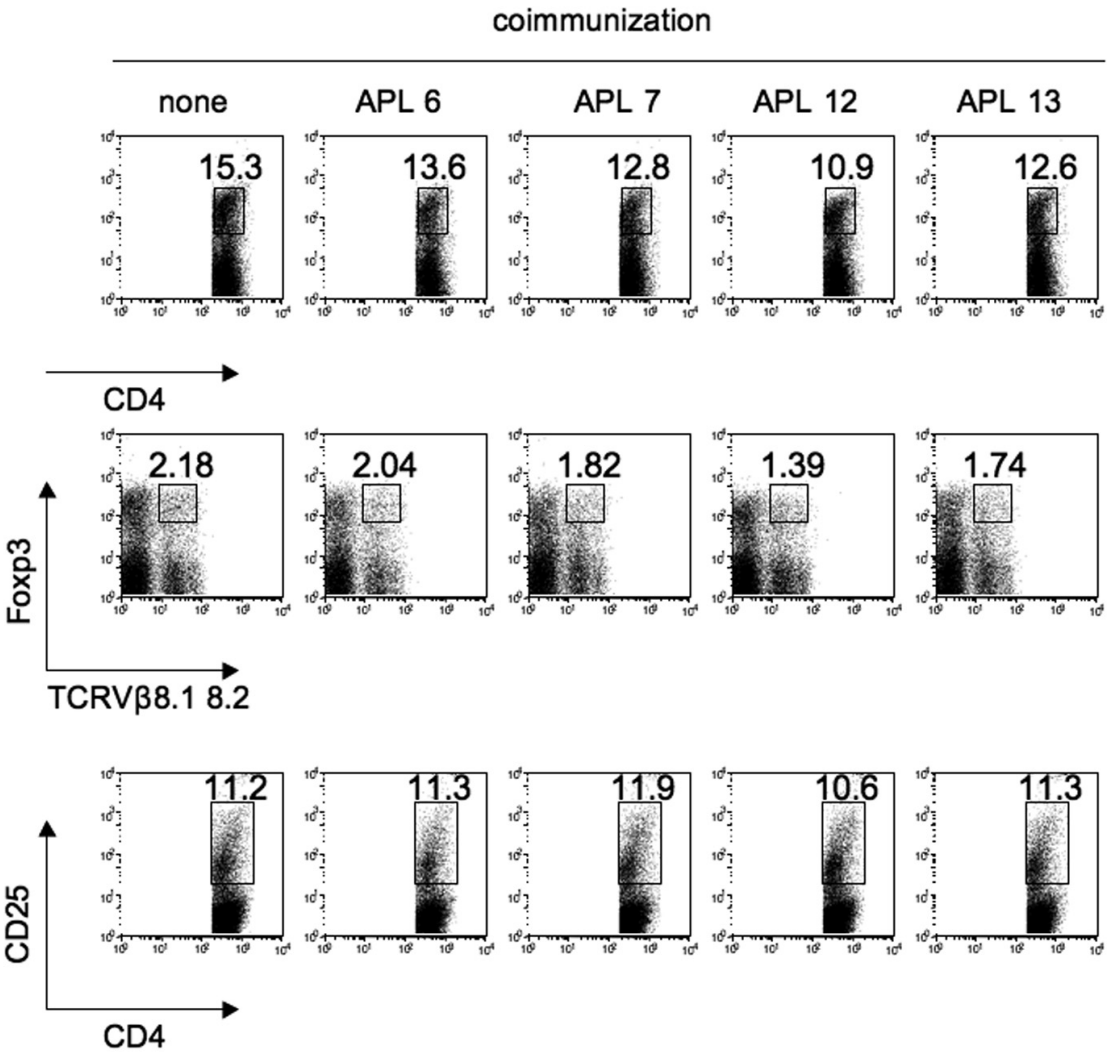
Co-immunization with altered peptide ligands neither induces regulatory T cells nor modulates CD25 expression. Mice were immunized with 10 μg glucose-6-phosphate isomerase hGPI_325--339_ and 50 μg each altered peptide ligand (APL), and draining lymph node cells on day 6 were stained with Foxp3 and CD25. Flow cytometry analysis was gated in CD4^+ ^cells. None, immunization with no APL (hGPI_325--339_ alone). Representative flow cytometry data of two independent experiments.

*In vitro *analysis showed that the antagonistic APLs suppressed IL-17 production, and that co-immunization with the APLs inhibited the development of arthritis. We therefore explored the effect of APLs on Th17 differentiation. Our previous report suggested that cross-reactivity of CD4^+ ^T cells primed with hGPI_325-339 _to mGPI_325-339 _was critical for the development of arthritis. We therefore assessed the population of mGPI_325-339 _reactive Th17 cells in the DLNs of mice co-immunized with each APL. IL-17 production by CD4^+ ^T cells with TCRVβ8.1 8.2 or other TCRVβ with stimulation of mGPI_325-339_was not affected by co-immunization with APLs (Figure 4a), and neither was affected IL-17 production with hGPI_325-339 _(data not shown). Unexpectedly, IL-17 production was considerable with stimulation of the corresponding APLs (Figure 4b). ELISA showed undetectable levels of IL-4, and the IL-10 production, and IFNγ production was not affected (data not shown). Co-immunization with APLs did neither affect the population of regulatory T cells nor the expression of CD25 (Figure 5), and stimulation of DLN cells of co-immunized mice with APLs *in vitro *did not induce the expansion of regulatory T cells (data not shown).

### Identification of TCRVβ usage of altered peptide ligand-specific CD4^+ ^T cells

**Figure 6:**
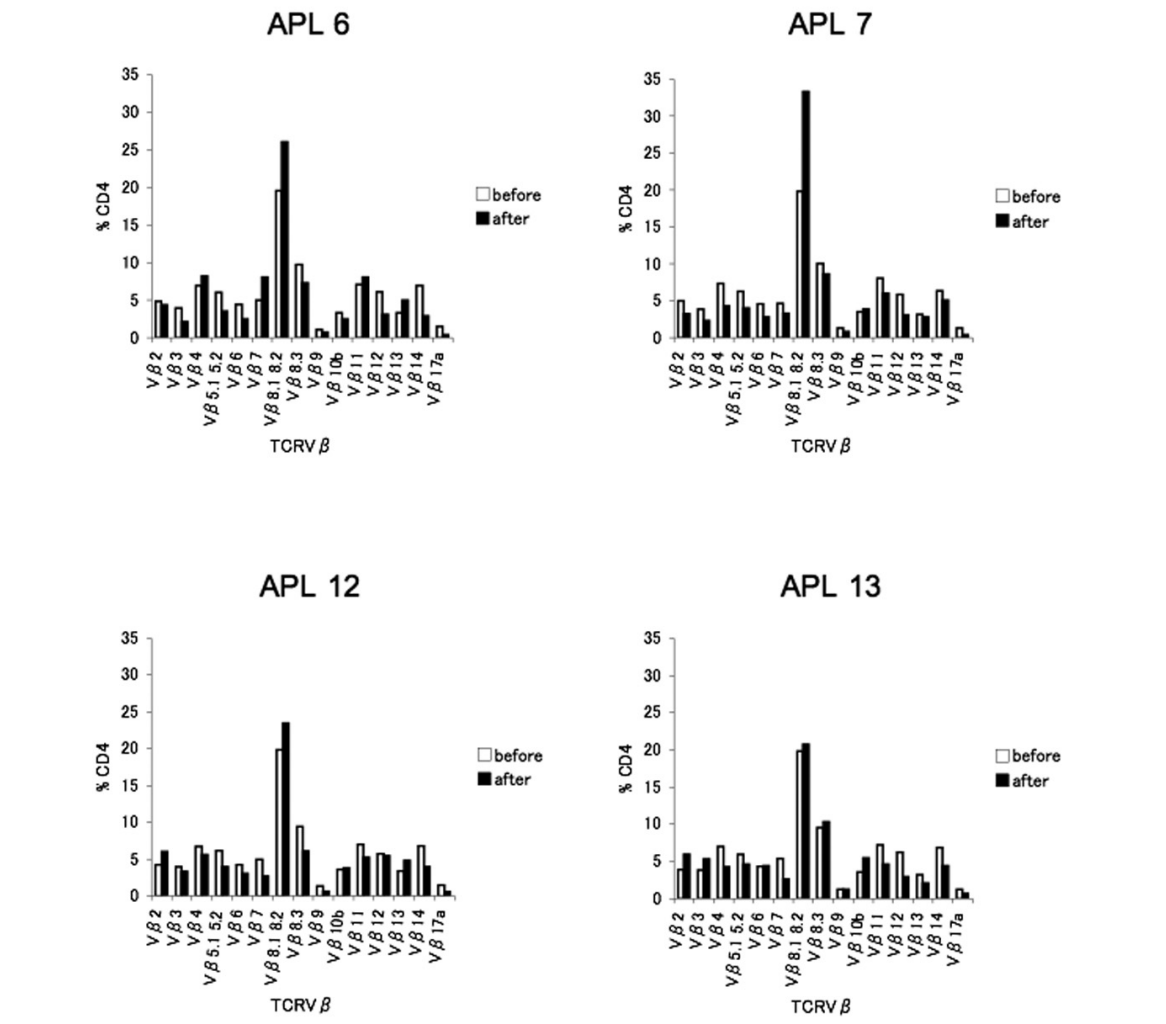
TCRVβ usage of altered peptide ligand-specific CD4^+ ^T cells. TCRVβ usage of altered peptide ligand (APL)-specific CD4^+ ^T cells was not remarkably shifted to TCRVβ8.1 8.2. Mice were immunized with 10 μg each APL, and draining lymph node cells on days 7 to 9 were stimulated with 20 μM corresponding APL *in vitro*. Ratios of TCRVβ repertoire to CD4^+ ^T cells. The TCRVβ repertoire of CD4^+ ^T cells was analyzed by flow cytometry: before stimulation with the corresponding *in vitro *for 72 hours, and after stimulation.

The unexpected data mentioned above suggested that APL-specific CD4^+ ^T cells were developed by co-immunization. We therefore investigated TCRVβ usage of APL-specific CD4^+ ^T cells. The CD4^+ ^T cells primed with each APL were stimulated with the corresponding APL *in vitro *and the TCRVβ repertoire was analyzed by flow cytometry and compared with that before stimulation. Stimulation with APL 6, APL 7 and APL 12 induced expansion of the population of CD4^+ ^T cells with TCRVβ8.1 8.2; however, this expansion was not so remarkable as that of hGPI_325-339_-specific CD4^+ ^T cells (Figures 3a and 6). Interestingly, stimulation with APL 13 hardly induced the expansion of the population of CD4^+ ^T cells with TCRVβ8.1 8.2 (Figure 6) or any other specific Vβ chain, although each APL stimulation could proliferate CD4^+ ^T cells primed with the corresponding APL as efficiently as hGPI_325-339 _(data not shown).

### Effects of antagonistic altered peptide ligands on anti-mouse glucose-6-phosphate isomerase antibody production

**Figure 7:**
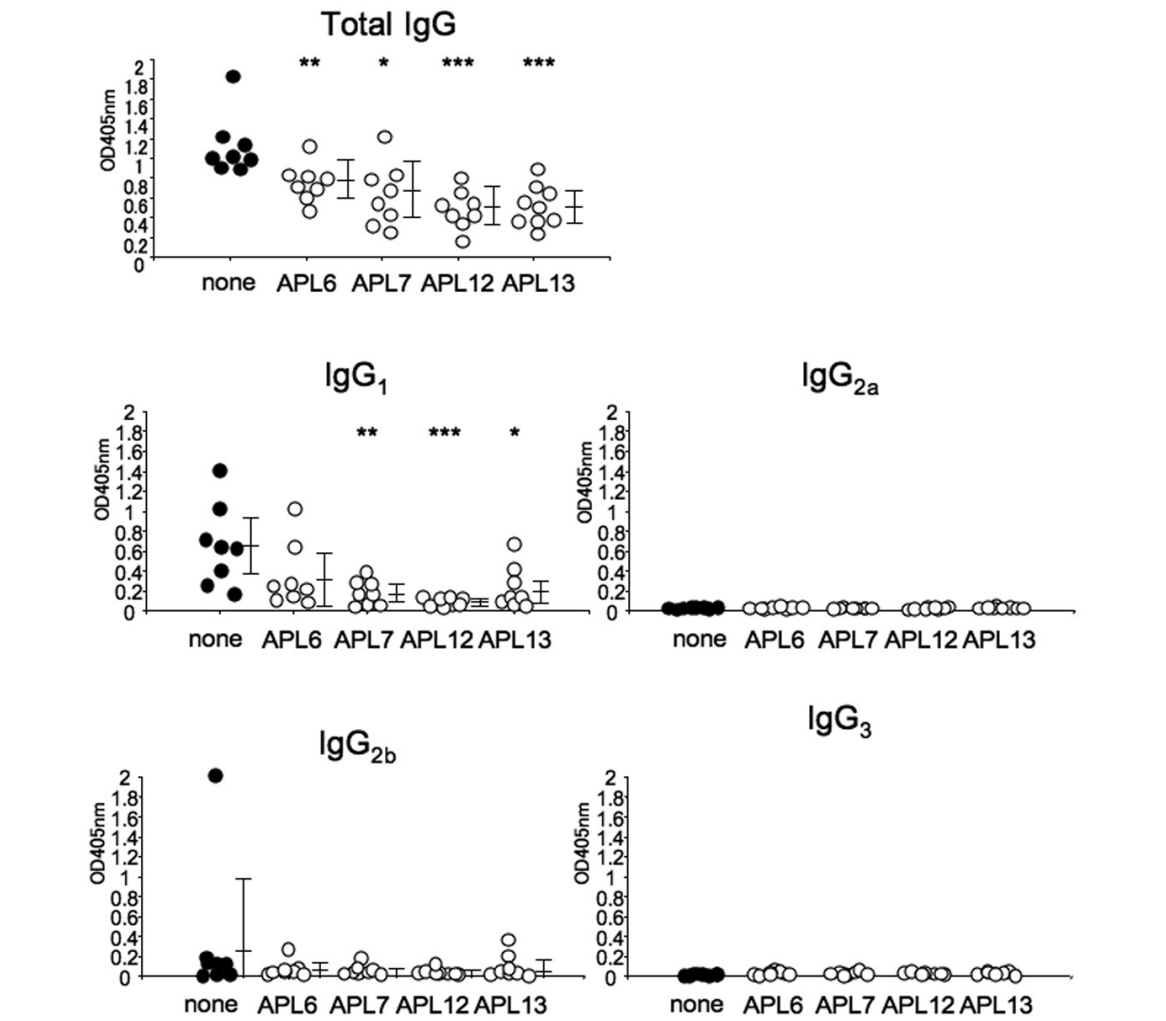
Co-immunization with altered peptide ligands suppresses production of antibodies to mouse glucose-6-phosphate isomerase. Sera were taken on day 28 from mice co-immunized with 10 μg glucose-6-phosphate isomerase hGPI_325--339_ and 50 μg each altered peptide ligand (APL), and the titers of anti-mouse GPI IgG and IgG isotype were analyzed by ELISA. Each symbol represents a single mouse. Data presented as mean optimal density ± standard deviation. *P < 0.01, **P < 0.005, ***P < 0.001 (Mann--Whitney U test).

Since administration of anti-CD4 monoclonal Abs with immunization prevents the development of arthritis and completely inhibits the production of anti-mGPI Abs in GPI-induced arthritis, mGPI is considered a thymus-dependent antigen to the humoral immune response [26]. We therefore next investigated the effects of APLs on antibody production. Co-immunization with APL 6, APL 7, APL 12 and APL 13 significantly suppressed the titers of anti-mGPI Abs (*P *< 0.01, *P *< 0.005, *P *< 0.001 and *P *< 0.001, respectively) (Figure 7). We also investigated the anti-mGPI IgG isotype. Co-immunization with APL 7, APL 12 and APL 13 significantly suppressed the titer of anti-mGPI IgG_1 _isotype (*P *< 0.005, *P *< 0.001 and *P *< 0.01, respectively). Any other anti-mGPI IgG isotype was hardly detected, however, and any bias to specific isotype was not found as an effect of APL.

## Discussion

APLs are considered useful for antigen-specific therapy of autoimmune diseases and allergy. Treatments with APLs have so far been tested in several autoimmune models, and especially experimental autoimmune encephalitis has been enthusiastically examined for APLs designed as a single amino acid substitution of a TCR contact site. In experimental autoimmune encephalitis in Lewis rats, co-immunization with the APL (K91A) of myelin basic protein MBP_87-99 _strongly inhibited the development of the disease by suppression of IFNγ and TNFα, not T-cell proliferation [31]. Furthermore, another study of experimental autoimmune encephalitis in SJL mice showed that co-immunization with the APL (W144Q) of myelin proteolipid protein PLP_139-151 _also inhibited the disease, and that the T-cell clone specific for the APL (W144Q) possessed the Th0 or Th2 phenotype [32].

Although one study used conventional APLs in collagen-induced arthritis [33], unconventional APLs with substitutions at MHC binding sites were mainly tested in arthritis models. Myers and colleagues reported that the analog peptide (I260A, A261B(hydroxyproline), F263N) significantly suppressed collagen-induced arthritis by inducing Th2 cells in DBA/1 mice [34]. They also reported the suppression of collagen-induced arthritis in HLA-DR1 and HLA-DR4 transgenic mice using other analog peptides with substitutions at MHC binding sites [35,36]. Another group reported also that the antigen-specific proinflammatory response to the human cartilage glycoprotein-39 (263 to 275) epitope was suppressed in DR4 transgenic mice by APLs with substitution at MHC binding sites [37].

In our study, we designed various APLs (N329S, N329T, G332A, or G332V) of hGPI_325-339 _with substitutions at TCR contact sites, and showed that co-immunization with the individual APL significantly inhibited the development of arthritis. Although the APLs had antagonism to Th17 primed with hGPI cells *in vitro *(Figure 1), analysis of the mechanisms of the effect of co-immunizing APLs showed normal development of hGPI-_325-339_-specific Th17 cells and APL-specific Th17 cells *in vivo *(Figure 4). Co-immunization with hGPI_325-339 _and the APL might have induced both hGPI-_325-339_-specific Th17 clones and APL-specific Th17 clones by the adjuvant effects of complete Freund's adjuvant and pertussis toxin.

Since both the TCR signal and the co-stimulatory signal are essential for priming of naïve T cells, our data suggested the potency of the APLs as agonists to some TCRs. It is likely that an antigen acts as an agonist to one T-cell clone and as an antagonist to another T-cell clone because any TCR can cross-react with various antigens. Although the antigen specificity is mainly determined by the complementary determining regions, the different ratio of TCRVβ usage between hGPI_325-339_-specific CD4^+ ^T cells and APL-specific CD4^+ ^T cells (especially APL 13) indicates that each CD4^+ ^T cell is a different clone that leads to different antigen specificity, and does not cross-react to the APLs and hGPI_325-339 _to conduct positive TCR signals, respectively. Our previous paper showed that T cells primed with hGPI_325-339 _could cross-react to mGPI_325-339 _and that the cross-reactivity to mGPI_325-339 _was crucial for induction arthritis [27]. The findings that immunization with the APLs (APL 6, APL 7, APL 12, APL 13) alone could not induce any overt arthritis indicated that APL-specific T cells could not cross-react mGPI_325-339 _suggesting they do not have potential for induction of arthritis.

One of the inhibitory mechanisms of APL is anergy. Allen and colleagues reported that APL could induce anergy of T-cell clones by partial activation [38], which was characterized by an increase in cell volume and upregulation of CD25, without cytokine production or cell proliferation. Another mechanism is induction of anti-inflammatory T-cell lineages such as Th2/Th0 as well as regulatory T cells. Nicholson and colleagues reported that co-immunization with PLP_139-151 _and APL (W144L/H147R) did not inhibit the induction of PLP_139-151_-specific T cells, but induced APL-specific Th2/Th0 phenotype cells to suppress the progression of experimental autoimmune encephalitis by stander suppression [39].

In our system, however, neither of these mechanisms was likely because the APLs did not inhibit IL-17 production and cell proliferation with stimulation of mGPI_325-339_, and induction of any anti-inflammatory and regulatory T cells was not detected. Nevertheless, it is probable that APLs inhibit mGPI_325-339_-specific T cells because our analysis showed significant reductions of anti-mGPI Abs, which were Abs to thymus-dependent antigen [26]. We assumed that competitive bindings of APL to TCR *in vivo *were likely in our system; however, it cannot be denied that amino acid substitutions in peptides, even those that are not directly involved in MHC binding, might affect the overall structure of the peptide and biding affinity to MHC. Taken together, competitive binding of the APLs to hGPI_325-339_-specific TCR or MHC *in vivo *is considered most likely as an inhibitory mechanism of APLs in our system.

The major interest in APLs is their clinical application; several studies showed that APLs suppress autoreactive cells in RA and Sjogren's syndrome [40,41]. Although clinical trials of APL in RA have not yet been conducted, phase II clinical trials in multiple sclerosis have been reported [42,43]. In one study of eight patients with multiple sclerosis, subcutaneous administration at 50 mg dose once-weekly of CGP77116 - an APL with substitutions at multiple TCR contact sites of MBP_83-99 _- resulted in the development of exacerbations in two patients with enhancement of MBP_83-99_-reactive Th1 response [42]. Another double-blind placebo-controlled clinical trial included 142 patients who received various doses of subcutaneously injected NBI5788, an APL of MBP_83-99 _with substitutions at TCR contact sites [43]. In contrast to the former study, the administration of 5 mg APL weekly significantly decreased inflammatory lesions in the central nervous system. Unfortunately, the study was halted because 9% of the patients developed hypersensitivity reactions, but none discontinued at a dose of 5 mg in the double-blind phase whereas all patients discontinued in the double-blind phase after receiving higher doses of 20 or 50 mg. Low-dose APLs might therefore be useful agents for antigen-specific therapies of autoimmune diseases including RA, and their efficacy in RA might be more promising than in multiple sclerosis because drugs can be injected directly into the inflammatory lesions.

Finally, can GPI be a target of antigen-specific therapies in RA? It has been reported that patients with severe forms of RA retained high titers of anti-GPI Abs [44-47] and GPI-reactive CD4^+ ^T cells were detected among anti-GPI-Ab-positive patients with RA [48]. These findings highlight autoimmune responses to GPI are occurring in some patients with RA, and GPI can be a target of antigen-specific therapies to them - although further studies are needed to clarify the exact pathological role of GPI in RA.

## Conclusions

The results of the present study showed that APLs with substitutions at TCR contact sites inhibit GPI peptide-induced arthritis. Novel antigen-specific therapies based on APLs may prove beneficial in arthritis induced by autoimmune responses to autoantigens.

## Abbreviations

Ab: antibody; APC: antigen-presenting cell; APL: altered peptide ligand; CII: collagen type II; DLN: draining lymph node; ELISA: enzyme-linked immunosorbent assay; GPI: glucose-6-phosphate isomerase; IFN: interferon; IL: interleukin; MBP: myelin basic protein; MHC: major histocompatibility complex; PBS: phosphate-buffered saline; PLP: proteolipid protein; RA: rheumatoid arthritis; rhGPI: recombinant human glucose-6-phosphate isomerase; TCR: T-cell receptor; Th: T-helper; TNF: tumor necrosis factor.

## Competing interests

The authors declare that they have no competing interests.

## Authors' contributions

KI wrote the manuscript and conducted all experiments. YY, AI, YK, KY YT, and RM assisted in the completion of the experiments. TS designed and coordinated the study. IM coordinated and directed the study. YN designed the APLs and provided advice for the study. TH, DG and SI participated in the discussion.
